# Synergistic Protective Effects of *Haematococcus pluvialis*-Derived Astaxanthin and Walnut Shell Polyphenols Against Particulate Matter (PM)_2.5_-Induced Pulmonary Inflammation

**DOI:** 10.3390/md23120473

**Published:** 2025-12-10

**Authors:** Hyun Kang, Jae-Ho Choi, Sung-Gyu Lee

**Affiliations:** 1 Department of Medical Laboratory Science, College of Health Science, Dankook University, Cheonan-si 31116, Republic of Korea; hkang@dankook.ac.kr (H.K.); smile_kogi@naver.com (J.-H.C.); 2 Marine Bio-Food and Drug Convergence Technology Center, Dankook University, Cheonan-si 31116, Republic of Korea

**Keywords:** *Haematococcus pluvialis*, microalga, astaxanthin, quercetin, COX-2, pulmonary inflammation

## Abstract

Airborne particulate matter (PM) triggers oxidative stress and inflammation in pulmonary tissues, contributing to chronic respiratory diseases. This study evaluated the antioxidant and anti-inflammatory effects of a combined extract of *Haematococcus pluvialis* (*H. pluvialis)* and walnut shell (HW extract) and its protective efficacy against PM_2.5_-induced pulmonary inflammation. Extracts mixed at different ratios (10:0–0:10, *w*/*w*) were tested using 2,2′-azino-bis(3-ethylbenzothiazoline-6-sulfonic acid) (ABTS) radical scavenging, cell-based assays, HPLC quantification, molecular docking, and a PM_2.5_-induced pulmonary inflammation mouse model. The optimized 6:4 mixture showed the strongest antioxidant activity (RC_50_ = 0.61 ± 0.14 μg/mL) and significantly reduced nitric oxide (NO) and cyclooxygenase-2 (COX-2) expression without cytotoxicity. HPLC confirmed the presence of astaxanthin (1.714 μg/mg) and quercetin (0.722 μg/mg). Docking simulations indicated strong COX-2 binding affinities (−9.501 and −8.753 kcal/mol) through hydrogen bonding and hydrophobic interactions. In vivo, HW extract reduced leukocyte infiltration, serum IL-6 levels, and pulmonary expression of COX-2, interleukin-6 (IL-6), and tumor necrosis factor-alpha (TNF-α) while improving alveolar structure. These results suggest that HW extract exerts synergistic antioxidant and anti-inflammatory actions via dual-site COX-2 modulation, providing a promising natural therapeutic approach for mitigating PM_2.5_-induced respiratory inflammation.

## 1. Introduction

Airborne particulate matter smaller than 2.5 μm in diameter (PM_2.5_) is a major air pollutant that penetrates deep into the alveoli, inducing oxidative stress, epithelial injury, and inflammation and thus leading to chronic respiratory disorders such as asthma, chronic obstructive pulmonary disease (COPD), and pulmonary fibrosis [1,2,3]. Mechanistically, PM_2.5_ exposure elevates intracellular reactive oxygen species (ROS) and activates redox-sensitive transcription factors such as nuclear factor-kappa B (NF-κB) and activator protein-1 (AP-1), which upregulate inflammatory mediators including cyclooxygenase-2 (COX-2), interleukin-6 (IL-6), and tumor necrosis factor-alpha (TNF-α) [4,5,6]. Chronic activation of these pathways contributes to tissue remodeling and irreversible pulmonary dysfunction [7,8].

A variety of therapeutic strategies have also been investigated for PM_2.5_-induced respiratory injury. Antioxidant agents such as N-acetylcysteine (NAC) have been reported to attenuate ROS-mediated neutrophil and monocyte recruitment and reduce pulmonary inflammation [9]. Recently, Brinsupri received regulatory approval (2025) for human use as an anti-inflammatory therapeutic for airway injury [10]. Conventional non-steroidal anti-inflammatory drugs (NSAIDs), including ibuprofen and naproxen, are sometimes used to alleviate inflammatory symptoms [11], while several natural compounds—such as vitamin D, melatonin, curcumin, omega-3 fatty acids, bixin, cirsilineol, EGCG, and metformin—have shown protective effects through modulation of oxidative stress, cytokine signaling, or epithelial barrier stabilization [12,13]. Celecoxib is cited in this study as a representative selective COX-2 inhibitor to illustrate the limitations of single-site inhibition compared with the multitarget synergistic potential of quercetin and astaxanthin.

Among these natural resources, *Haematococcus pluvialis* (*H. pluvialis*), a freshwater microalga widely used in the marine biotechnology and aquaculture industries, and *Juglans regia* (walnut shell) represent highly complementary sources of polyphenolic and carotenoid antioxidants [14,15,16]. *H. pluvialis* is known as the most abundant natural source of astaxanthin, a xanthophyll carotenoid that potently scavenges singlet oxygen and lipid radicals while stabilizing cellular membranes [17,18,19]. Numerous studies have reported that astaxanthin attenuates oxidative damage and inflammation by activating Nuclear factor erythroid 2–related factor 2/Heme oxygenase-1 (Nrf2/HO-1) signaling and suppressing NF-κB and mitogen-activated protein kinase (MAPK) pathways [20,21,22,23]. In parallel, walnut shells, often treated as agro-industrial by-products, are rich in quercetin and ellagic acid polyphenols with demonstrated COX-2 inhibitory and cytokine-suppressive activities [24,25,26,27]. Quercetin directly binds to the COX-2 catalytic pocket, forming hydrogen bonds with residues such as Arg120, Tyr355, and Ser530 [28], while also regulating IL-6 and TNF-α transcription through NF-κB inhibition [29,30].

Recently, computational and structural biology studies have revealed that bioactive molecules with distinct binding regions on COX-2 or 5-Lipoxygenase (LOX) may act synergistically through dual-site or allosteric modulation [31,32,33,34,35]. Quercetin’s polar scaffold favors hydrogen bonding within the catalytic site, whereas astaxanthin’s elongated hydrophobic chain enables interaction with peripheral or allosteric sites [36,37]. This complementary binding pattern may potentiate enzyme inhibition and reduce off-target toxicity compared with single-site synthetic inhibitors such as celecoxib [38]. However, no prior research has systematically evaluated the combinatorial efficacy of *H. pluvialis* and walnut shell extracts in oxidative or inflammatory pulmonary injury models.

Accordingly, this study aimed to (i) optimize the ratio of *H. pluvialis* and walnut shell extracts for maximal bioactivity, (ii) elucidate the molecular interactions of astaxanthin and quercetin with COX-2 using in silico docking analyses, and (iii) verify the in vivo efficacy of the optimized formulation against PM_2.5_-induced pulmonary inflammation. By integrating biochemical, computational, and histopathological approaches, this work highlights a dual-site enzyme inhibition mechanism as a promising strategy for designing natural multi-component therapeutics targeting respiratory inflammation [39,40,41,42].

## 2. Results

### 2.1. Optimization of Extract Ratio

#### 2.1.1. ABTS Radical Scavenging Activity

**Figure 1:**
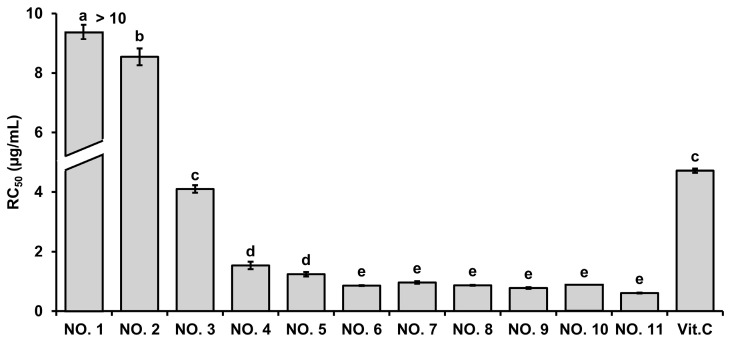
ABTS radical scavenging activity of *H. pluvialis* and walnut shell extracts and their mixtures at different ratios (10:0 to 0:10, *w*/*w*). Values are expressed as mean ± SD (*n* = 3). Radical scavenging efficacy was compared based on RC_50_ values (concentration required to scavenge 50% of ABTS radicals). The bar for NO. 1 is truncated to indicate that the RC_50_ value exceeded the Y-axis range, and the actual value (> 10 μg/mL) is labeled above the bar. Different letters (a–e) above the bars indicate significant differences at *p* < 0.05, as determined by one-way ANOVA followed by Tukey’s post hoc test.

The antioxidant potential of *H. pluvialis* extract, walnut shell extract, and their mixtures was evaluated by the ABTS radical scavenging assay, and the results are expressed in terms of RC_50_ values (Figure 1). The *H. pluvialis* extract alone (NO. 1) showed negligible scavenging activity, with an RC_50_ value exceeding 10 μg/mL, indicating poor efficacy. To clearly reflect this in the visualization, the NO. 1 bar in Figure 1 is truncated, and the actual value (>10 μg/mL) is annotated above the bar. In contrast, walnut shell extract alone (NO. 11) exhibited potent activity, with an RC_50_ value of 0.61 ± 0.14 μg/mL. Interestingly, mixtures of *H. pluvialis* and walnut shell extracts showed a ratio-dependent enhancement of antioxidant activity. Formulations containing ≥ 30% walnut shell extract (NO. 4–NO. 11) displayed markedly lower RC_50_ values (0.61–1.53 μg/mL), indicating strong radical scavenging capacity. Among them, NO. 6–NO. 10 (ratios 5:5 to 1:9) demonstrated the most potent effects, with RC_50_ values significantly lower than that of vitamin C (4.72 ± 0.25 μg/mL, positive control), confirming superior antioxidant efficacy (*p* < 0.05).

#### 2.1.2. Cytotoxicity

**Figure 2:**
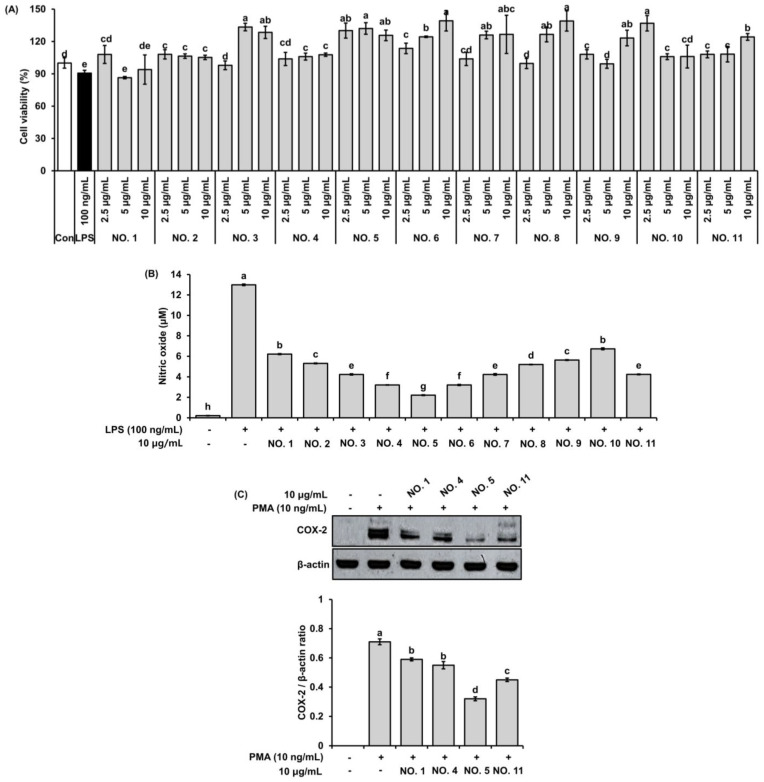
Anti-inflammatory effects of *H. pluvialis*, walnut shell extracts, and their mixtures in vitro. (**A**) Cell viability of RAW 264.7 macrophages treated with different ratios of extracts (2.5, 5, and 10 μg/mL) for 24 h, as assessed by the MTT assay. (**B**) Inhibitory effects of extracts (10 μg/mL) on nitric oxide (NO) production in LPS (100 ng/mL)-stimulated RAW 264.7 macrophages. (**C**) Inhibitory effects of selected extracts (10 μg/mL) on COX-2 protein expression in PMA (10 ng/mL)-stimulated A549 airway epithelial cells, analyzed by Western blotting and quantified as the COX-2/β-actin ratio. Values are expressed as mean ± SD (*n* = 3). Different letters (a–g) above the bars indicate significant differences at *p* < 0.05, as determined by one-way ANOVA followed by Tukey’s post hoc test.

The cytotoxicity of *H. pluvialis*, walnut shell extracts, and their mixtures was evaluated in RAW 264.7 macrophages using the MTT assay (Figure 2A). LPS stimulation significantly reduced cell viability compared with the untreated control group. Treatment with extract mixtures (2.5–10 μg/mL) restored cell viability in a dose-dependent manner. None of the tested extracts exhibited cytotoxic effects. In particular, mixtures containing walnut shell extract (NO. 2–NO. 11) showed significantly higher cell viability compared with the LPS group (*p* < 0.05).

#### 2.1.3. Inhibitory Effect on NO Production

The effect of the extracts on NO production was investigated in LPS-stimulated RAW 264.7 macrophages (Figure 2B). LPS markedly increased NO production (12.9 μM), while treatment with extract mixtures (10 μg/mL) significantly inhibited NO accumulation in a ratio-dependent manner. The mixtures generally showed stronger inhibitory effects than the single extracts, with NO. 4–NO. 6 (ratios 7:3 to 5:5) exhibiting the most pronounced activity, reducing NO levels to 3–5 μM (*p* < 0.05).

#### 2.1.4. Suppression of COX-2 Protein Expression in Pulmonary Epithelial Cells

To further confirm the anti-inflammatory effects, two extract mixtures that showed superior antioxidant and anti-inflammatory activities in previous assays were selected and compared with the individual extracts. COX-2 protein expression was evaluated in PMA-stimulated A549 pulmonary epithelial cells as an in vitro model to assess their potential as therapeutic agents for alleviating pulmonary inflammation (Figure 2C). PMA treatment strongly upregulated COX-2 expression, whereas pretreatment with extracts (10 μg/mL) markedly suppressed its induction. Notably, the selected mixtures, particularly NO. 5 (6:4 ratio), exhibited more pronounced inhibitory effects than the single extracts, reducing the COX-2/β-actin ratio by more than 50% compared with PMA-treated cells (*p* < 0.05).

### 2.2. Quantification of Marker Compounds in the Selected Extract Mixture

**Figure 3:**
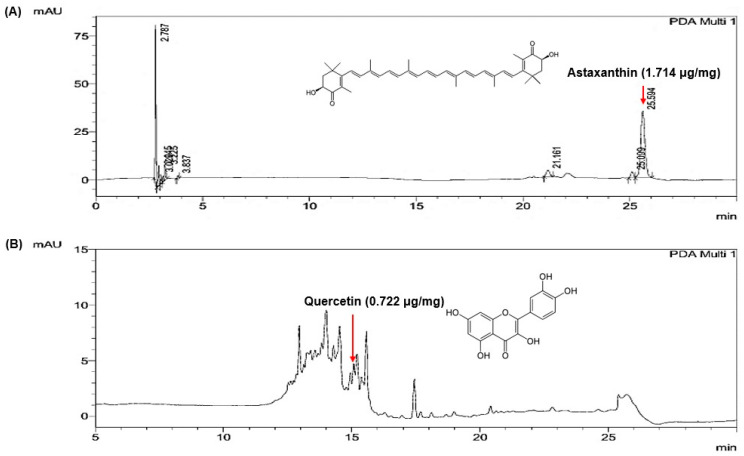
Quantitative HPLC chromatograms of marker compounds in the optimized extract mixture (NO. 5, *H. pluvialis*/walnut shell = 6:4, *w*/*w*). Representative HPLC chromatograms of (**A**) astaxanthin detected at 474 nm and (**B**) quercetin detected at 334 nm.

Quantitative HPLC analysis was conducted to determine the levels of the marker compounds in the optimized extract mixture (NO. 5; *H. pluvialis*/walnut shell = 6:4, *w*/*w*). As shown in Figure 3, distinct peaks corresponding to astaxanthin and quercetin were identified in the chromatograms of the mixed extract. The astaxanthin peak was detected at a retention time of 25.53 min at 474 nm (Figure 3A), and the quercetin peak appeared at 14.87 min at 334 nm (Figure 3B). The contents of astaxanthin and quercetin in the mixture were quantified as 1.714 μg/mg and 0.722 μg/mg, respectively.

In addition to marker compound quantification, total flavonoid content (TFC) and total polyphenol content (TPC) of the optimized extract were newly determined using standard colorimetric assays. The TFC and TPC values, summarized in Table 1, confirm that quercetin represents only a minor fraction of the overall flavonoid pool and that the extract is rich in diverse polyphenolic constituents. These results support the interpretation that the observed biological effects arise from the combined action of multiple polyphenols rather than from quercetin alone.

### 2.3. Molecular Docking Analysis of Quercetin and Astaxanthin with COX-2

#### 2.3.1. Ligand and Protein Preparation

**Figure 4:**
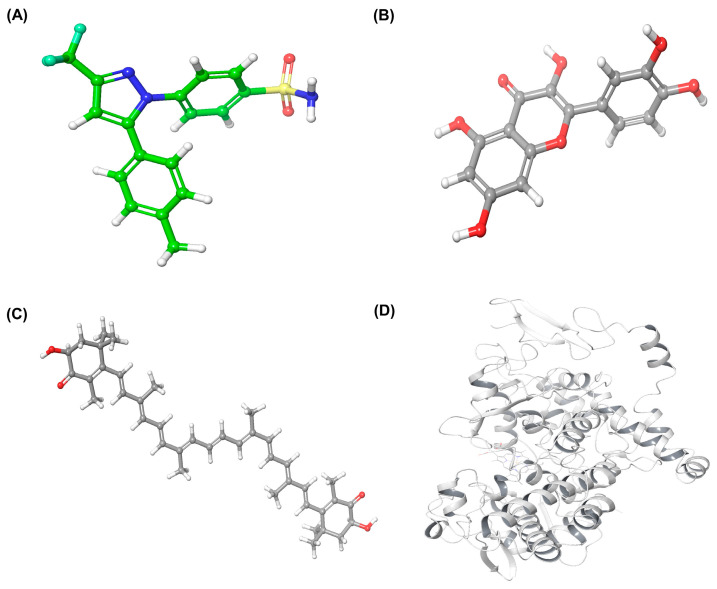
Prepared structures used for molecular docking. (**A**) Celecoxib, (**B**) Quercetin, (**C**) Astaxanthin, and (**D**) the optimized COX-2 protein (PDB ID: 3LN1) generated using the Protein Preparation Wizard in Schrödinger.

The 3D structures of celecoxib, quercetin, and astaxanthin were successfully prepared using the LigPrep module of Schrödinger under the OPLS4 force field at physiological pH (7.4). Each ligand was energy-minimized and converted to its most stable conformation. The COX-2 enzyme structure (PDB ID: 3LN1) was retrieved from the RCSB Protein Data Bank and optimized using the Protein Preparation Wizard to correct missing atoms, assign bond orders, optimize hydrogen-bond networks, and remove unnecessary water molecules. The prepared ligands and protein structures are presented in Figure 4.

#### 2.3.2. Identification of Binding Sites

**Figure 5:**
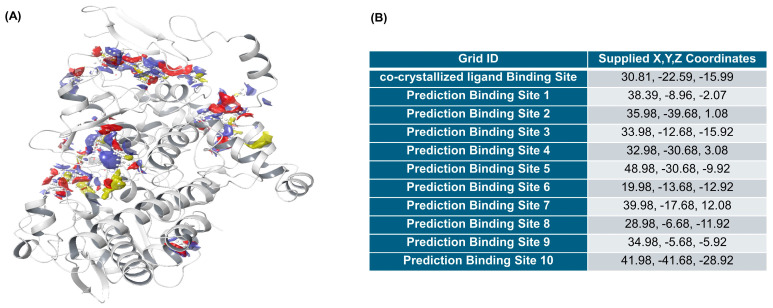
Predicted binding sites and grid generation for COX-2 (PDB: 3LN1). (**A**) Visualization of predicted potential binding pockets identified using the SiteMap module in Schrödinger, highlighted in color on the prepared COX-2 protein structure. (**B**) Grid generation summary showing the supplied X, Y, and Z coordinates for the co-crystallized ligand binding site and ten predicted binding sites used for molecular docking simulations.

The potential binding pockets of COX-2 were predicted using the SiteMap module of Schrödinger. A total of 10 binding pockets were identified in addition to the native celecoxib binding site (Figure 5A). These regions were used to generate docking grids, with coordinates summarized in Figure 5B.

#### 2.3.3. Docking Validation

The docking protocol was validated by re-docking the co-crystallized ligand celecoxib into its native active site. The reproduced poses yielded RMSD values ranging from 0.46 Å to 7.02 Å (Table 2). The presence of multiple conformations with RMSD < 2.0 Å confirmed the reliability and accuracy of the docking parameters.

#### 2.3.4. Molecular Docking and Interaction Analysis

**Figure 6:**
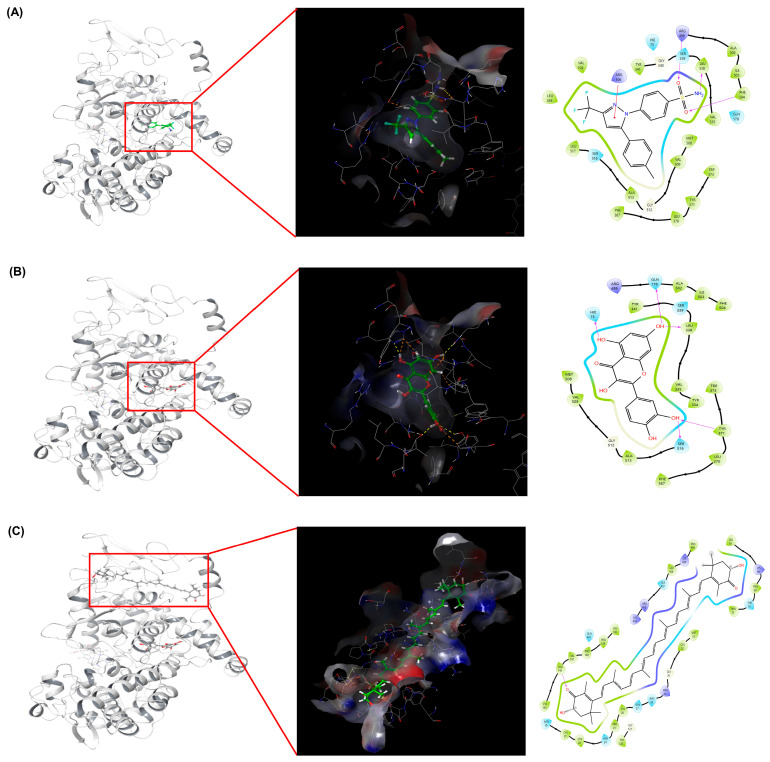
Binding interaction analysis of COX-2 with docked ligands. Overall 3D and 2D representations of the best docking poses showing the binding conformations of (**A**) celecoxib, (**B**) quercetin, and (**C**) astaxanthin within the COX-2 binding pocket. Each panel illustrates (left) the overall protein structure highlighting the ligand-binding region, (middle) the 3D interaction map depicting hydrophobic and polar surface contacts, and (right) the 2D schematic representation of hydrogen bonds and hydrophobic interactions with key amino acid residues.

Molecular docking simulations were performed to evaluate the binding affinities and interaction profiles of celecoxib, quercetin, and astaxanthin against the COX-2 enzyme (PDB: 3LN1). Among the tested ligands, celecoxib exhibited the most favorable docking score (−11.427 kcal/mol) within the native active pocket, consistent with its known inhibitory activity. Quercetin also displayed strong affinity (−9.501 kcal/mol) at the same catalytic region, forming hydrogen bonds with key residues Hie75, Gln178, Leu338, Tyr371, and Ser516. Astaxanthin showed the best docking score (−8.753 kcal/mol) at a predicted binding site (Site 1) identified by the SiteMap module, located near the hydrophobic pocket adjacent to the membrane-binding domain. Figure 6 depicts the representative 3D and 2D interaction maps for each ligand.

### 2.4. Protective Effects of HW Extracts Against PM_2.5_-Induced Pulmonary Inflammation in Mice

#### 2.4.1. BALF Cell Analysis

**Figure 7:**
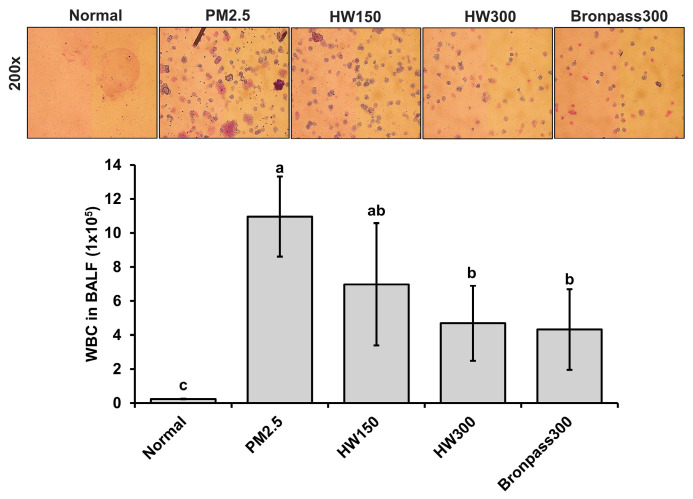
Effects of HW extracts on total WBC counts in BALF of PM_2.5_-induced pulmonary inflammation in mice. Representative microscopic images (200× magnification) showing inflammatory cell infiltration in BALF from each group (Normal, PM_2.5_, HW150, HW300, and Bronpass300). Values are expressed as mean ± SD (*n* = 5). Different letters (a–c) above the bars indicate statistically significant differences at *p* < 0.05 according to one-way ANOVA followed by Tukey’s post hoc test. Enlarged high-resolution images of panels are provided in Supplementary Figure S1.

Exposure to PM_2.5_ significantly increased inflammatory cell infiltration in BALF, indicating an acute inflammatory response in the pulmonary. The WBC count in BALF of the PM_2.5_-exposed group reached approximately 10.84 × 10^5^ cells, representing a 45-fold increase compared with the normal control (0.24 × 10^5^ cells). Administration of HW extracts markedly reduced WBC counts in a dose-dependent manner: 6.82 × 10^5^ cells in the HW150 group and 4.10 × 10^5^ cells in the HW300 group, corresponding to 37% and 62% reductions, respectively, relative to the PM_2.5_ group. The positive-control Bronpass300 group exhibited a comparable suppressive effect (3.82 × 10^5^ cells) (Figure 7). These findings indicate that HW extract effectively prevents inflammatory cell infiltration into the airway lumen following PM_2.5_ exposure.

#### 2.4.2. Serum IL-6 Analysis

**Figure 8:**
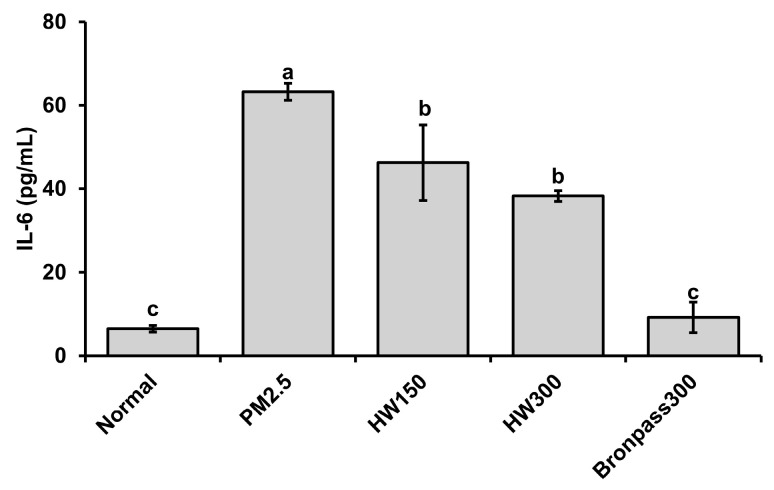
Effects of HW extracts on IL-6 levels in serum of PM_2.5_-induced pulmonary inflammation in mice. Values are expressed as mean ± SD (n = 5). Different letters (a–c) above the bars indicate statistically significant differences at *p* < 0.05 according to one-way ANOVA followed by Tukey’s post hoc test.

PM_2.5_ exposure markedly increased IL-6 concentration in BALF to 65.24 pg/mL, demonstrating a strong systemic inflammatory response. Treatment with HW extracts significantly attenuated this elevation, reducing IL-6 levels to 45.79 pg/mL in the HW150 group and 38.91 pg/mL in the HW300 group. The Bronpass300 group exhibited near-normal IL-6 concentrations (7.46 pg/mL), indicating potent inhibition of cytokine overproduction (Figure 8). These results suggest that HW extract, particularly at 300 mg/kg, effectively minimizes PM_2.5_-induced cytokine secretion and inflammatory signaling.

#### 2.4.3. Pulmonary Tissue Analysis (RT-PCR and Histopathology)

**Figure 9:**
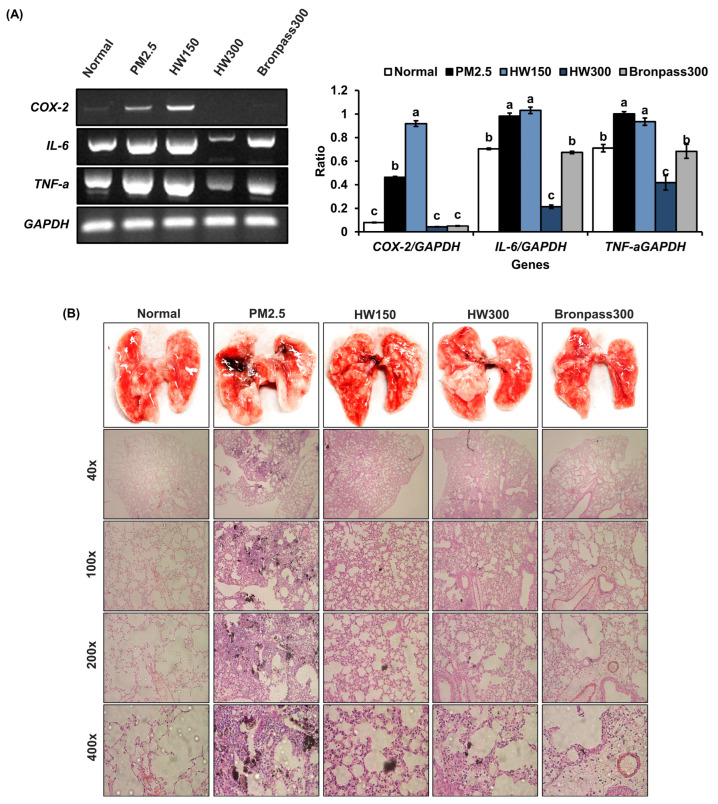
Effects of HW extracts on inflammatory gene expression and histopathological changes in pulmonary tissue of PM_2.5_-induced pulmonary inflammation in mice. (**A**) RT-PCR analysis of pro-inflammatory genes in pulmonary tissue. (**B**) Histopathological examination of pulmonary tissues using gross morphology and H&E-stained sections at different magnifications (40×, 100×, 200×, and 400×). Values are expressed as mean ± SD (*n* = 5). Different letters (a–c) above the bars indicate statistically significant differences at *p* < 0.05 according to one-way ANOVA followed by Tukey’s post hoc test. Enlarged high-resolution images of panels are provided in Supplementary Figure S2.

RT-PCR analysis of pulmonary tissue revealed that PM_2.5_ exposure markedly upregulated the mRNA expression of *COX-2*, *IL-6*, and *TNF-α* by approximately 1.2–4.0 fold compared with the normal control, confirming a strong inflammatory response at the transcriptional level. In contrast, HW300 treatment significantly downregulated these pro-inflammatory genes, decreasing *COX-2* from 0.48 (PM_2.5_ group) to 0.08 (−83%), IL-6 to 0.28, and TNF-α to 0.42, comparable to those observed in the Bronpass300 group. The HW150 group showed only marginal suppression of these genes (Figure 9A). Histopathological evaluation further supported these molecular findings. The PM_2.5_ group exhibited severe alveolar wall thickening, hemorrhage, and dense infiltration of inflammatory cells, indicating extensive pulmonary injury. In contrast, HW-treated mice showed dose-dependent structural recovery, with HW300 markedly preserving alveolar integrity and reducing inflammatory lesions to a level similar to the positive-control group (Figure 9B). Collectively, these results confirm that HW extract, particularly at the higher dose, exerts potent protective effects against PM_2.5_-induced pulmonary inflammation by suppressing pro-inflammatory gene expression and preventing leukocyte accumulation in pulmonary tissue.

## 3. Discussion

The present study demonstrated that the combined extract of *H. pluvialis* and walnut shell (HW extract) exhibited potent antioxidant and anti-inflammatory effects, which contributed to its protective role against PM_2.5_-induced pulmonary inflammation. HW extract effectively reduced leukocyte infiltration, suppressed cytokine expression, and improved pulmonary histopathology. These findings support the hypothesis that combining carotenoid and polyphenol-rich extracts provides synergistic biological activity through complementary antioxidant and anti-inflammatory mechanisms.

The ABTS radical scavenging assay (Figure 1) demonstrated that the antioxidant activity of the extract mixtures increased proportionally with the walnut shell extract ratio. The optimized 6:4 formulation (NO. 5) exhibited markedly enhanced activity compared with the single extracts, suggesting a synergistic interaction between astaxanthin and polyphenolic constituents. In cell-based assays (Figure 2), the same mixture significantly reduced nitric oxide production and COX-2 protein expression without affecting cell viability, indicating anti-inflammatory activity independent of cytotoxicity. These results are in agreement with previous reports that *H. pluvialis*-derived astaxanthin reduces oxidative stress and cytokine expression in macrophage and epithelial models [43,44,45], and that walnut-derived polyphenols suppress NF-κB-mediated inflammatory responses [24,46,47]. HPLC quantification (Figure 3) confirmed the presence of astaxanthin (1.714 μg/mg) and quercetin (0.722 μg/mg) in the mixture. The coexistence of these two antioxidant classes—lipophilic and hydrophilic—may underlie the enhanced efficacy observed in both antioxidant and anti-inflammatory assays.

Because the HPLC method used in this study was not optimized for complete chromatographic separation of flavonoids and carotenoids, the quantification results (Figure 3) should be interpreted as crude-level, semi-quantitative profiling rather than precise compound-specific measurements. Accordingly, the detected quercetin value represents a marker component within the extract, and we do not claim it to be the principal active constituent. Instead, the observed biological activities are attributed to the combined action of multiple polyphenols and carotenoids naturally present in the crude extract.

However, despite the well-known antioxidant potency of astaxanthin, the *H. pluvialis* extract alone exhibited weak ABTS radical scavenging activity in this study. This outcome may be explained by the chemical nature of the extract, in which astaxanthin predominantly exists in esterified forms that possess lower radical-scavenging reactivity than free astaxanthin in certain in vitro systems [48,49]. Furthermore, the lipid-rich extraction matrix may reduce the solubility and assay accessibility of carotenoids, thereby diminishing measurable activity in aqueous ABTS assays [50]. These matrix-related interferences likely contribute to the high RC_50_ value observed for the *H. pluvialis* extract.

It is also important to note that the aim of this study was to evaluate the synergistic bioactivity of naturally co-occurring metabolites within crude extracts rather than isolated compounds. For this reason, purified astaxanthin was not employed. Nevertheless, future studies incorporating both free and esterified astaxanthin standards—as well as purified polyphenols—will help clarify the mechanistic contributions of individual constituents to the observed antioxidant and anti-inflammatory effects.

Molecular docking simulations using COX-2 (PDB: 3LN1) revealed that quercetin and astaxanthin exhibit strong binding affinities at or near the celecoxib binding pocket (Figure 6). Quercetin interacted with Hie75, Gln178, Leu338, Tyr371, and Ser516, while astaxanthin bound hydrophobic residues such as Ala142. These results, summarized in Table 3, provide supportive evidence for potential interactions between HW extract components and COX-2 rather than direct confirmation of catalytic modulation. The docking validation results (Figure 5) further support the reliability of the docking model. However, because individual quercetin and astaxanthin COX-2 catalytic inhibition assays were not performed, the docking outcomes should be interpreted as predictive and not as evidence of direct enzyme-level modulation. Although docking results do not directly confirm in vivo inhibition, the correlation between COX-2 docking scores and decreased COX-2 expression observed in A549 cells (Figure 2C) provides consistent mechanistic insight. Previous studies have similarly reported COX-2 suppression by quercetin [51,52,53] and astaxanthin [40,54,55], supporting the plausibility of the observed interactions, although further studies are required to determine whether these constituents directly inhibit COX-2 enzymatic activity.

In the murine PM_2.5_ exposure model, HW extract treatment resulted in a significant reduction in BALF leukocyte counts (Figure 7) and IL-6 levels (Figure 8), both key indicators of airway inflammation [56,57,58]. The HW300 group showed effects comparable to the positive-control group, suggesting a robust anti-inflammatory action. RT-PCR analysis and histopathology (Figure 9) confirmed that HW extract markedly decreased *COX-2*, *IL-6*, and *TNF-α* expression and improved alveolar integrity. These findings align with prior observations that PM_2.5_ exposure activates COX-2/NF-κB signaling leading to cytokine overproduction and tissue injury [6,59,60,61]. The reduction in inflammatory gene expression after HW treatment indicates suppression of these pathways, likely through the combined antioxidant and COX-2–inhibitory activities of its constituents.

Taken together, the data suggest that HW extract exerts its protective effects through dual antioxidant–anti-inflammatory synergy. Astaxanthin scavenges reactive oxygen species and stabilizes cellular membranes, while quercetin inhibits pro-inflammatory signaling and enzyme activation. The concurrent modulation of oxidative stress and cytokine expression explains the dose-dependent protective effects observed across Figure 1, Figure 2, Figure 3, Figure 4, Figure 5, Figure 6, Figure 7, Figure 8 and Figure 9. Such dual-action mechanisms are consistent with previous reports describing the efficacy of mixed natural antioxidants in mitigating air pollutant-induced pulmonary injury [12,13]. However, further investigations, including molecular pathway validation (e.g., NF-κB, Nrf2, MAPKs) and chronic exposure models, are warranted to fully characterize HW extract’s mechanism of action.

Importantly, our findings not only confirm the biological effectiveness of the combined extract but also highlight a broader therapeutic concept: natural products with complementary antioxidant and anti-inflammatory constituents may provide a multi-target strategy for mitigating air-pollution–related respiratory injury [9,12,13]. This concept is particularly relevant given the complex and multifactorial nature of PM_2.5_-induced pathology. Although a quantitative synergy coefficient was not calculated in the present study, both ABTS and NO inhibition data consistently demonstrated ratio-dependent potentiation, supporting the presence of functional synergy between astaxanthin and polyphenolic components [48,49]. Additionally, to prevent overinterpretation of the semi-quantitative HPLC data, conclusions regarding the role of quercetin were moderated, and synergy was evaluated at the extract level rather than on individual compounds.

Future work incorporating formal synergy quantification (e.g., Combination Index or Bliss Independence models) and mechanism-focused assays will be valuable to fully validate the synergistic interactions suggested by our data [62].

## 4. Materials and Methods

### 4.1. Materials

Astaxanthin standard (≥98%) and quercetin standard (≥95%) were purchased from Sigma-Aldrich (St. Louis, MO, USA). Phorbol 12-myristate 13-acetate (PMA), lipopolysaccharide (LPS, *Escherichia coli* O111:B4), 3-(4,5-dimethylthiazol-2-yl)-2,5-diphenyltetrazolium bromide (MTT), and dimethyl sulfoxide (DMSO) were also obtained from Sigma-Aldrich (St. Louis, MO, USA). Fetal bovine serum (FBS), Dulbecco’s Modified Eagle Medium (DMEM), and Roswell Park Memorial Institute (RPMI-1640) medium were purchased from Gibco (Thermo Fisher Scientific, Waltham, MA, USA). Antibodies against COX-2 and β-actin, and horseradish peroxidase (HRP)-conjugated secondary antibodies were obtained from Cell Signaling Technology (Danvers, MA, USA). Ethanol (analytical grade), acetonitrile, and methanol (HPLC grade) were supplied by Merck (Darmstadt, Germany). All other chemicals and reagents used were of analytical or cell culture grade and used as received without further purification.

### 4.2. Preparation of Extracts and Mixture Formulations

**Figure 10:**
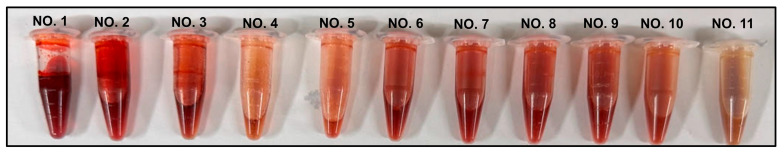
Representative images of extract mixtures of *H. pluvialis* and walnut shell at different mixing ratios (10:0 to 0:10, *w*/*w*). Sample numbers correspond to NO. 1–NO. 11, as shown from left to right.

The *H. pluvialis* extract was kindly provided by Yuhan Care Co., Ltd. (Seoul, Republic of Korea). Briefly, dried *H. pluvialis* biomass was pulverized using an air jet mill (80–100 psi) and subjected to supercritical CO_2_ extraction with ethanol as a co-solvent (5 mL/min) at 500–550 bar and 60 °C. The resulting crude extract was concentrated at 50 °C under reduced pressure, further purified to remove residual solvents and by-products, and standardized as *H. pluvialis* extract. The walnut shell (*Juglans regia*) extract was prepared in our laboratory. Dried walnut shells were pulverized into fine powder and extracted with 70% (*v*/*v*) ethanol under reflux conditions. The extract was filtered, concentrated under reduced pressure at 55 °C, and freeze-dried to yield a dry extract powder. For mixture preparation, the two extracts were blended at defined weight ratios of *H. pluvialis* to walnut shell (10:0, 9:1, 8:2, 7:3, 6:4, 5:5, 4:6, 3:7, 2:8, 1:9, and 0:10, *w*/*w*) to obtain a series of standardized formulations for subsequent experiments, as summarized in Table 4 and visually represented in Figure 10.

### 4.3. 2,2′-Azino-bis(3-ethylbenzothiazoline-6-sulfonic acid) (ABTS) Radical Scavenging Assay

The antioxidant activity of the extracts was determined using the ABTS radical scavenging assay, as previously described in our previous study [63] with minor modifications. Briefly, the ABTS•^+^ solution was generated by mixing 7 mM ABTS with 2.45 mM potassium persulfate and incubating the mixture in the dark at room temperature (RT) for 24 h. The solution was then diluted with ethanol to obtain an absorbance of 0.70 ± 0.02 at 732 nm. Extract samples at various concentrations were added to the ABTS•^+^ solution and incubated for 1 min in the dark. The absorbance was measured at 732 nm using a microplate reader (xMarkTM, BIO-RAD, Hercules, CA, USA). The radical scavenging activity was calculated as a percentage reduction in absorbance compared with the control. The radical scavenging concentration 50% (RC_50_) value, defined as the concentration of extract required to scavenge 50% of ABTS radicals, was calculated from the dose–response curve. Ascorbic acid (Vit. C) was used as a positive control.

### 4.4. Cell Culture

RAW 264.7 murine macrophages (Korean Cell Line Bank; KCLB, Seoul, Korea) and A549 human pulmonary epithelial cells (KCLB) were cultured under identical conditions at 37 °C in a humidified incubator containing 5% CO_2_. RAW 264.7 cells were maintained in DMEM supplemented with 10% FBS and 1% penicillin–streptomycin, while A549 cells were cultured in RRPMI-1640 containing the same supplements. Cells were subcultured at 70–80% confluence and used for experiments within 5–6 passages.

### 4.5. Cell Viability (MTT) Assay

Cell viability was determined in RAW 264.7 cells using the MTT assay as described previously with minor modifications [64]. Briefly, cells were seeded in 96-well plates (1 × 10^4^ cells/well) and treated with extract mixtures (2.5, 5, and 10 μg/mL) for 24 h. MTT solution (5 mg/mL, 20 μL/well) was added and incubated for 4 h; formazan crystals were dissolved in DMSO and absorbance was measured at 570 nm using a microplate reader. Viability was calculated as a percentage of the untreated control.

### 4.6. Nitric Oxide (NO) Production Assay

RAW 264.7 cells were pretreated with extract mixtures (10 μg/mL) for 1 h and stimulated with LPS (100 ng/mL) for 24 h. Nitrite levels in culture supernatants were quantified using the Griess reagent method as described previously [65] absorbance was read at 540 nm and concentrations were calculated from a sodium nitrite standard curve.

### 4.7. Western Blot Analysis

A549 cells were pretreated with samples for 1 h and then stimulated with PMA (10 ng/mL) for 24 h. Total proteins were extracted, separated by SDS-PAGE, and transferred to PVDF membranes. Membranes were probed with anti-COX-2 and anti-β-actin antibodies, followed by HRP-conjugated secondary antibodies. Bands were visualized by ECL and quantified (ImageJ 1.54i, NIH, Bethesda, MD, USA). COX-2 levels were normalized to β-actin.

### 4.8. HPLC Analysis of Marker Compounds

The quantitative analysis of quercetin and astaxanthin was performed using an HPLC system (LC-20AD, Shimadzu Scientific Instruments Inc., Kyoto, Japan) equipped with a ZORBAX SB-C18 column (5 µm, 4.6 × 250 mm; Agilent Technologies Inc., Santa Clara, CA, USA) and a UV–visible detector. The detection wavelengths were set at 334 nm for quercetin and 474 nm for astaxanthin. Although 480 nm is commonly used for free astaxanthin, preliminary UV–Vis spectral scanning of the extract dissolved in DMSO revealed a solvent- and matrix-dependent absorbance maximum at 474 nm. This blue-shift (3–6 nm) is consistent with the known spectral behavior of esterified astaxanthin species, which predominate in *H. pluvialis* extracts; therefore, 474 nm was selected to ensure accurate quantification under the chromatographic conditions used in this study. The mobile phase consisted of solvent A (0.1% phosphoric acid in distilled water) and solvent B (acetonitrile). The flow rate was maintained at 1.0 mL/min, and the column temperature was controlled at 40 °C. The injection volume was 20 µL for all samples and standards. Samples and standard compounds (quercetin and astaxanthin) were dissolved in DMSO before injection. The gradient elution was conducted as follows: the initial mobile phase composition was 95% solvent A and 5% solvent B, which was maintained for the first 5 min. The proportion of solvent B was then linearly increased to 100% over 15 min and held constant from 15 to 20 min. Subsequently, the gradient returned to the initial condition (95% A and 5% B) over 5 min and was maintained for an additional 5 min for re-equilibration. The total analysis time was 30 min.

### 4.9. Determination of Total Polyphenol and Total Flavonoid Contents

The TPC and TFC of the extracts were quantified using standard colorimetric assays with slight methodological adjustments. For TPC analysis, each extract (1 mg/mL) was mixed with an equal volume of Folin–Ciocalteu reagent that had been pre-diluted 1:1 with distilled water. After standing for 3 min at RT, 1 mL of 10% sodium carbonate solution was added to initiate chromophore development. The reaction was allowed to proceed for 1 h at RT, and absorbance was subsequently recorded at 700 nm using a microplate spectrophotometer (xMark™, Bio-Rad). Gallic acid served as the calibration standard, and TPC values were expressed as mg gallic acid equivalents per gram of extract (mg GAE/g). For TFC measurement, the method reported by Moreno et al. [66] was adapted. Briefly, 0.1 mL of extract was diluted with 0.9 mL of 80% ethanol, and 0.5 mL of this solution was combined with 10% aluminum nitrate (0.1 mL), 1 M potassium acetate (0.1 mL), and 4.3 mL of 80% ethanol. Following a 40 min incubation at RT, absorbance was measured at 415 nm. Quercetin was used to construct the standard curve, and TFC was expressed as mg quercetin equivalents per gram of extract (mg QE/g).

### 4.10. Molecular Modeling and Docking Simulation

All molecular modeling, protein–ligand docking, and interaction analyses were performed using the Schrödinger Suite (Maestro version 14.3.129, MMshare version 6.9.129, Release 2025-1, Platform: Windows-x64).

#### 4.10.1. Ligand Preparation

The molecular structures of quercetin (CID: 5280343) and astaxanthin (CID: 5281224) were obtained from the PubChem database (https://pubchem.ncbi.nlm.nih.gov/, accessed on 10 September 2025) in SMILES format. The SMILES strings were converted into 3D structures using the 2D Sketcher in Maestro. The ligands were preprocessed using the LigPrep module with the OPLS4 force field, and the pH was set to 7.4 ± 0.5. Up to 500 possible conformers were generated to ensure structural diversity. The co-crystallized ligand celecoxib was included for docking protocol validation.

#### 4.10.2. Protein Preparation

The 3D crystal structure of cyclooxygenase-2 (COX-2, PDB ID: 3LN1) was retrieved from the RCSB Protein Data Bank (https://www.rcsb.org/, accessed on 10 September 2025). All heteroatoms, water molecules, and redundant chains were removed, except for the essential heme (HEM) cofactor. The Protein Preparation Wizard was employed to optimize the structure, assign protonation states, and minimize energy using the OPLS4 force field. Hydrogen bonds were optimized and missing side chains were corrected to ensure stereochemical integrity.

#### 4.10.3. Grid Generation

The receptor grid was generated around the binding pocket of the native inhibitor (celecoxib). The SiteMap module was used to predict potential binding pockets, selecting up to five top-ranked sites. Any site exceeding a volume of 800 Å^3^ was subdivided into sub-sites. A grid box size of 25 Å was defined based on the molecular dimensions of astaxanthin.

#### 4.10.4. Ligand Docking

Docking simulations were performed using the Glide module in Standard Precision (SP) mode. The receptor was treated as rigid, while ligand flexibility was fully allowed. The docking score was used as the main criterion for binding affinity. Sequential docking was carried out to investigate dual-site interactions: ligands were first docked into the native celecoxib binding site, followed by docking into predicted secondary binding sites. The co-crystallized ligand celecoxib was re-docked to verify grid accuracy and validate docking parameters.

#### 4.10.5. Ligand–Protein Interaction Analysis

Ligand–protein interactions were analyzed using the Ligand Interaction Diagram tool in Schrödinger. Two-dimensional (2D) and three-dimensional (3D) interaction maps were generated to visualize hydrogen bonds, hydrophobic contacts, π–π stacking, and other non-covalent interactions. Key amino acid residues involved in stabilizing the ligand within the COX-2 active site were identified and compared among ligands.

### 4.11. Evaluation of Antioxidant and Anti-Inflammatory Effects in a PM_2.5_-Induced Pulmonary Inflammation Mouse Model

#### 4.11.1. PM_2.5_-Induced Pulmonary Inflammation Model and Experimental Design

Particulate matter (PM_2.5_; Standard Reference Material 1648a) was purchased from Sigma-Aldrich (St. Louis, MO, USA). This certified reference material is widely used in air pollution research due to its well-defined and stable composition. For animal experiments, PM_2.5_ was suspended in phosphate-buffered saline (PBS) at a dose of 50 mg/kg. Male BALB/c mice (6 weeks old, 20 ± 2 g) were obtained from DaeHan BioLink Co., Ltd. (Eumseong, Chungbuk, Korea) and maintained under controlled environmental conditions (23 ± 2 °C, relative humidity 50 ± 5%, 12 h light/dark cycle) with free access to standard chow and water. After a one-week acclimatization period, the mice were randomly divided into five groups (*n* = 5 per group): (1) Normal control, (2) PM_2.5_-exposed group, (3) PM_2.5_ + HW extract (*H. pluvialis*/walnut shell = 6:4, *w*/*w*) 150 mg/kg (HW150), (4) PM_2.5_ + HW extract 300 mg/kg (HW300), and (5) PM_2.5_ + Bronpass Tablet 300 mg/kg (Bronpass300, positive control; Hanlim Pharm. Co., Yongin, Korea). The HW extract and Bronpass Tablet were dissolved in distilled water and orally administered once daily for eight consecutive days. On day 7, pulmonary inflammation was induced by intratracheal instillation of PM_2.5_ (50 mg/kg, 50 µL). On day 8, all mice were anesthetized with isoflurane and sacrificed for sample collection. Bronchoalveolar lavage fluid (BALF), blood, and pulmonary tissues were collected for biochemical and histological analyses. All animal procedures were conducted in accordance with the guidelines of the Institutional Animal Care and Use Committee (IACUC) of Dankook University (approval number: DKU-25-049).

#### 4.11.2. Total White Blood Cell Count in BALF

BALF samples were collected by flushing the pulmonary three times with sterile PBS. The recovered lavage fluid was centrifuged to pellet the cells, and red blood cells were lysed using RBC lysis buffer. The remaining cells were resuspended in PBS, and total white blood cell (WBC) counts were determined using methylene blue staining and a hemocytometer. For morphological examination, cytospin preparations were stained with Wright–Giemsa solution to visualize infiltrated inflammatory cells.

#### 4.11.3. Measurement of Serum IL-6 Levels

Serum IL-6 levels were measured using a commercial ELISA kit in accordance with the manufacturer’s protocol. Each serum sample was analyzed in duplicate, and absorbance was recorded at 450 nm using a microplate reader (Bio-Rad, Hercules, CA, USA). The IL-6 concentrations were calculated from a standard curve generated with recombinant IL-6 standards and expressed as pg/mL.

#### 4.11.4. Histopathological Examination of Pulmonary Tissue (H&E Staining)

Pulmonary tissues were fixed in 10% neutral-buffered formalin, embedded in paraffin, and sectioned at a thickness of 5 μm. The tissue sections were stained with hematoxylin and eosin (H&E) and examined under a light microscope (Olympus, Tokyo, Japan) to assess histopathological changes in pulmonary morphology.

#### 4.11.5. Analysis of Inflammatory Gene Expression in Pulmonary Tissue (RT-PCR)

Total RNA was extracted from frozen pulmonary tissues using TRIzol reagent (Thermo Fisher Scientific, Waltham, MA, USA) according to the manufacturer’s protocol. RNA purity and concentration were determined spectrophotometrically at 260 and 280 nm using a NanoDrop Lite spectrophotometer (Thermo Fisher Scientific). For complementary DNA (cDNA) synthesis, 1 µg of total RNA was reverse-transcribed using a RT premix kit (Bioneer, Daejeon, Republic of Korea). PCR amplification was conducted using a commercial PCR premix (Bioneer) in a total reaction volume of 20 µL containing cDNA template and 10 pmol of each primer. The thermal cycling conditions were: 94 °C for 5 min; 40 cycles of 94 °C for 30 s, 58 °C for 30 s, and 72 °C for 60 s; followed by a final extension at 72 °C for 5 min. PCR products were electrophoresed on 1.5% agarose gels containing ethidium bromide and visualized under UV light using a Gel Doc™ EZ Imager (Bio-Rad). Band intensities were quantified using ImageJ software, and target gene expression levels were normalized to GAPDH. The primer sequences used for amplification are listed as follows: COX-2: forward 5′-ACATCCCTGAGAACCTGCAGT-3′, reverse 5′-CCAGGAGGATGGAGTTGTTGT-3′; IL-6: forward 5′-GGAGGCTTAAITACACATGTT-3′, reverse 5′-TGATTCAAGATGAATTGGAT-3′; TNF-α: forward 5′-TTCGAGTGACAAGCCTGTAGC-3′, reverse 5′-AGATTGACCTCAGCGCTGAGT-3′; GAPDH: forward 5′-CCAGTATGACTCCACTCACG-3′, reverse 5′-CCTTCCACAATGCCAAGTT-3′.

### 4.12. Statistical Analysis

All experiments were performed in triplicate, and the results are expressed as mean ± standard deviation (SD). Statistical significance among groups was evaluated using one-way analysis of variance (ANOVA), followed by Tukey’s multiple comparison post hoc test. A value of *p* < 0.05 was considered statistically significant.

## 5. Conclusions

The combined extract of *H. pluvialis* and walnut shell (HW extract) exhibited strong antioxidant and anti-inflammatory effects that collectively protected against PM_2.5_-induced pulmonary inflammation. The optimized 6:4 formulation showed synergistic radical scavenging and COX-2-inhibitory activities, supported by the presence of astaxanthin and quercetin and their favorable COX-2 binding interactions. In vivo, HW extract significantly reduced leukocyte infiltration, IL-6 secretion, and inflammatory gene expression while improving pulmonary histopathology. These findings suggest that HW extract exerts dual antioxidant and anti-inflammatory actions, providing a promising natural therapeutic strategy for preventing air pollution-related respiratory inflammation.

## Figures and Tables

**Table 1:** Total polyphenol content and total flavonoid content of the optimized extract mixture (NO. 5, *H. pluvialis*/walnut shell = 6:4, *w*/*w*).

Parameter	Contents
Total Polyphenol Content (TPC)	1185.49 ± 20.35 mg GAE/g ^1,3^
Total Flavonoid Content (TFC)	56.29 ± 11.87 mg QE/g ^2,3^

^1^ TPC was measured using the Folin–Ciocalteu method and expressed as mg gallic acid equivalent (GAE) per g extract. ^2^ TFC was determined using the aluminum chloride colorimetric method and expressed as mg quercetin equivalent (QE) per g extract. ^3^ Data are presented as mean ± SD (*n* = 3)

**Table 2:** Validation of docking protocol: RMSD of re-docked celecoxib poses.

Celecoxib Pose ID	RMSD (Å)	Celecoxib Pose ID	RMSD (Å)
1	0.4610	13	6.1187
2	0.6477	14	6.8377
3	0.8432	15	5.1316
4	0.9605	16	6.1564
5	4.9750	17	7.0174
6	0.8623	18	5.1730
7	5.5747	19	6.8061
8	0.9668	20	6.9348
9	5.5523	21	6.8886
10	6.0899	22	6.5071
11	5.5572	23	1.7841
12	6.1417		

**Table 3:** Molecular docking summary of celecoxib, quercetin, and astaxanthin with COX-2.

Ligand	Binding Site ID	Docking Score (kcal/mol)	Key Interacting Residues	Type of Interaction
Celecoxib	Co-crystallized active site	−11.427	Arg499, Leu338, Phe504, Arg106	Hydrogen bonding, π–π stacking
Quercetin	Co-crystallized active site	−9.501	Hie75, Gln178, Leu338, Tyr371, Ser516	Hydrogen bonding
Astaxanthin	Predicted binding site 1	−8.753	Ala142	Hydrogen bonding

**Table 4:** Mixing ratios of *H. pluvialis* and walnut shell extracts.

Sample NO.	NO. 1	NO. 2	NO. 3	NO. 4	NO. 5	NO. 6	NO. 7	NO. 8	NO. 9	NO. 10	NO. 11
Ratio (H ^1^:W ^2^)	10:0	9:1	8:2	7:3	6:4	5:5	4:6	3:7	2:8	1:9	0:10

^1^ *H. pluvialis* extracts; ^2^ Walnut shell extracts.

## Data Availability

The datasets used and/or analyzed during the current study are available from the corresponding author upon request.

## Supplementary Materials

The following supporting information can be downloaded at https://www.mdpi.com/article/10.3390/md23120473/s1, Figure S1: Representative BALF cytology images showing the effects of HW extract on PM_2.5_-induced pulmonary inflammation; Figure S2: Gross pulmonary morphology and H&E-stained histopathology showing the protective effects of HW extract against PM_2.5_-induced pulmonary injury.

## Abbreviations

The following abbreviations are used in this manuscript:

ROS	Reactive oxygen species
NF-κB	Nuclear factor-kappa B
AP-1	Linear dichroism
COX-2	Cyclooxygenase-2
IL-6	Interleukin-6
TNF-α	Tumor necrosis factor-alpha
Nrf2	Nuclear factor erythroid 2–related factor 2
HO-1	Heme oxygenase-1
LOX	Lipoxygenase
MAPK	Mitogen-activated protein kinase
LPS	Lipopolysaccharide
PMA	Phorbol 12-myristate 13-acetate
BALF	Bronchoalveolar lavage fluid
