# Supplementary figures and images for: Synergistic Protective Effects of Haematococcus pluvialis-Derived Astaxanthin and Walnut Shell Polyphenols Against Particulate Matter (PM)2.5-Induced Pulmonary Inflammation
Source: Mar Drugs. 2025 Dec 10;23(12):473. doi: 10.3390/md23120473 (PMC12734818; doi:10.3390/md23120473)

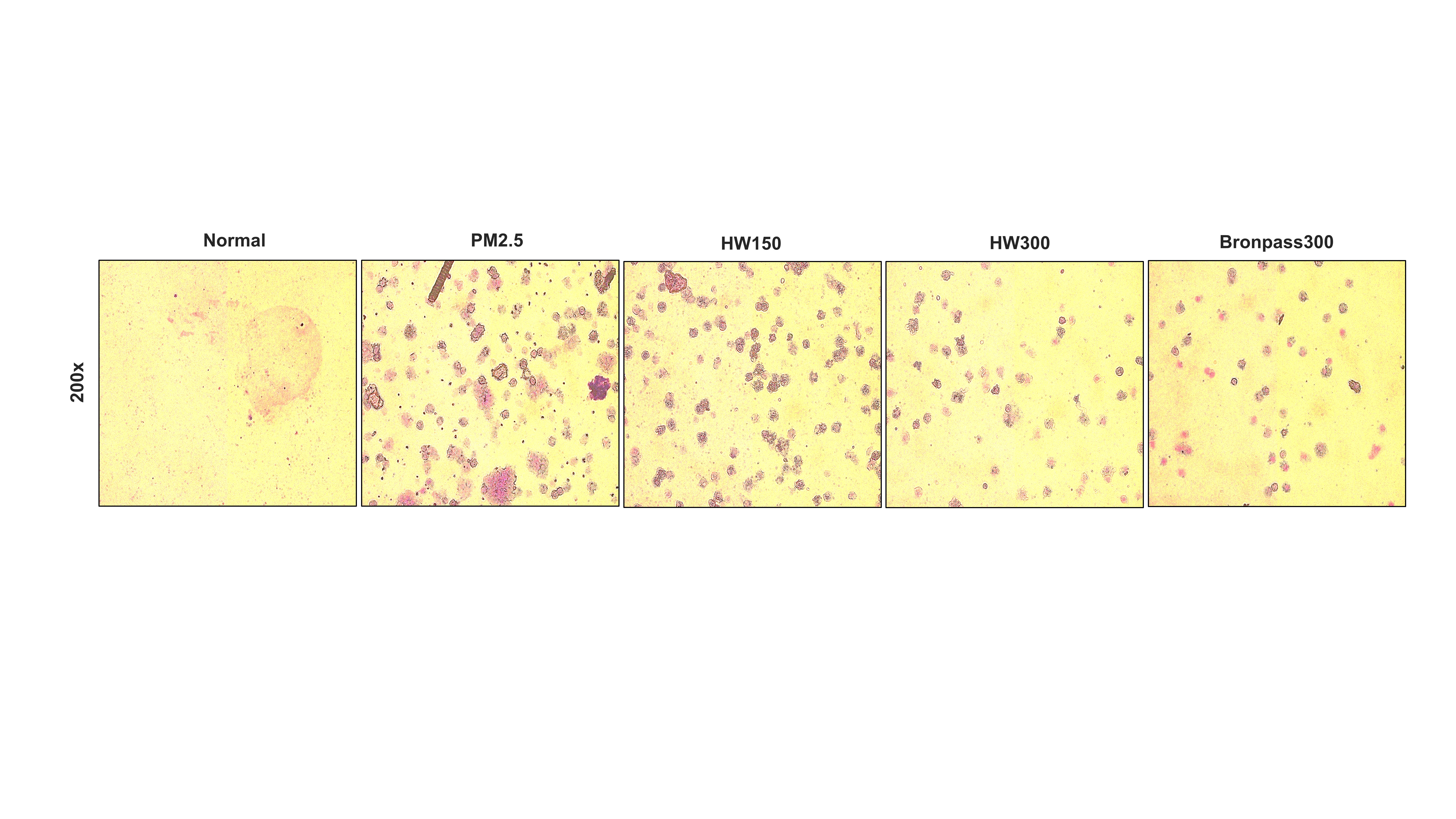

Supplement: Supplementary file 1 [file marinedrugs-23-00473-s001.zip › Supplementary file/Supplementary Fig. S1. .jpg]

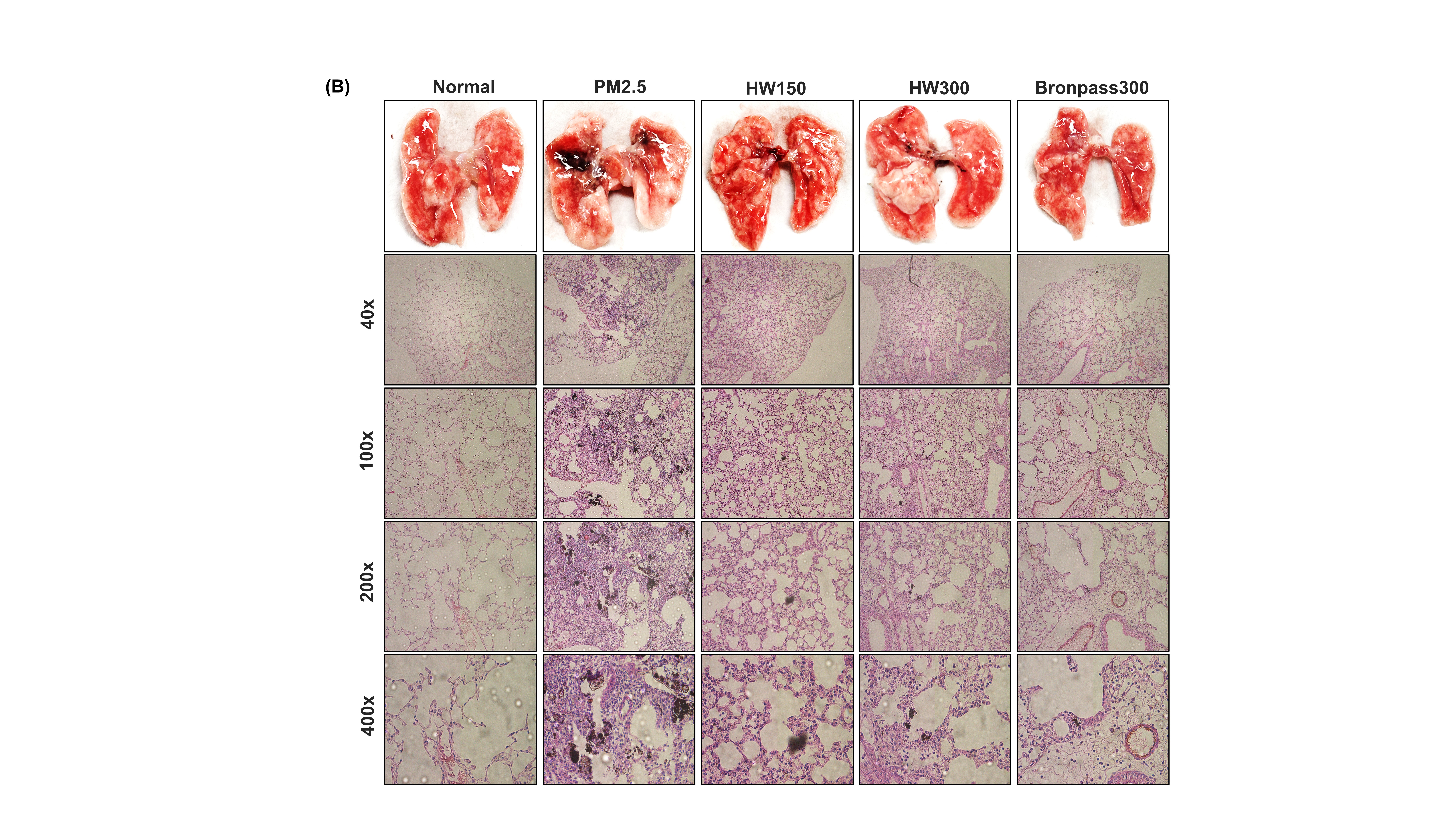

Supplement: Supplementary file 1 [file marinedrugs-23-00473-s001.zip › Supplementary file/Supplementary Fig. S2..jpg]
